# Development of selective deconjugases for membrane-anchored LC3A/B in post-mitotic neurons

**DOI:** 10.1186/s13041-025-01184-z

**Published:** 2025-02-12

**Authors:** Haneul Choi, Sang-Won Park, Deok-Jin Jang, Jin-A Lee

**Affiliations:** 1 Department of Biological Sciences and Biotechnology, College of Life Sciences and Nanotechnology, Hannam University, Daejeon, Korea; 2 Department of Vector Entomology, College of Ecology and Environment, Kyungpook National University, Sangju, Korea; 3 Department of Ecological Science, College of Ecology and Environment, Kyungpook National University, Sangju, Korea

**Keywords:** LC3/GABARAP, Neuronal autophagy, Deconjugases, RavZ

## Abstract

Neuronal autophagy is essential for maintaining protein and organelle turnover, thereby safeguarding neuronal health. LC3, a central autophagy protein, exists in lipidated (LC3-II) and non-lipidated (LC3-I) forms, both critical for neurons due to their sensitivity to metabolic and proteostatic stress. To elucidate the specific roles of membrane-anchored LC3A/B in post-mitotic neurons, we engineered deconjugases with enhanced selectivity for lipidated LC3. By modifying LC3-interacting regions (LIRs) at the deconjugase termini, we significantly improved targeting specificity toward LC3A/B. Deconjugases with N-terminal LIR modifications reduced LC3A/B-associated autophagosomes, highlighting the importance of LIR positioning for specificity. Sequential N-terminal LIR arrangements further refined LC3A/B targeting without affecting GABARAP-associated autophagosomes. Moreover, reducing the hydrophobicity of the α3 helix to limit membrane residence time further improved selectivity. These targeted modifications demonstrate the potential of customized deconjugases to dissect and modulate specific autophagic pathways in neurons, paving the way for novel therapeutic strategies against neurodegenerative diseases associated with autophagy dysregulation.

## Main text

Autophagy is a tightly regulated process essential for degrading damaged cellular components within double-membrane autophagosomes [1]. In post-mitotic neurons, it plays a critical role in maintaining cellular homeostasis and long-term survival, as neurons cannot dilute damaged components through division [2, 3, 4]. Autophagy is vital for removing misfolded proteins, damaged organelles, and debris, whose accumulation can lead to dysfunction and neurodegenerative diseases like Alzheimer’s, Parkinson’s, and Huntington’s [5].

During autophagy, LC3/GABARAP proteins undergo lipidation, where cytosolic LC3-I/GABARAP-I is conjugated to phosphatidylethanolamine (PE) on autophagosomal membranes, forming LC3/GABARAP-II. This ATG7-ATG3-mediated process, akin to ubiquitination, is crucial for autophagosome formation and cargo recruitment. Beyond autophagy, LC3/GABARAP proteins regulate membrane dynamics, including elongation, closure, vesicle trafficking, and organelle maintenance, while also participating in non-canonical pathways like LC3-associated phagocytosis, linking innate immunity and membrane remodeling [6]. Although LC3 and GABARAP family proteins exist in two forms—lipidated (LC3/GABARAP-II) and non-lipidated (LC3/GABARAP-I)—the lack of technologies to selectively inhibit LC3/GABARAP-II has, until recently, hindered the study of membrane-anchored LC3/GABARAP functions in post-mitotic neuron. We developed an irreversible deconjugase that delipidates LC3/GABARAP-II on membranes using LIR motifs and the CAD of *Legionella pneumophila* RavZ protein [7]. In this study, we enhanced the selectivity of deconjugases_Fy for LC3A/B to investigate the role of membrane-anchored LC3A/B in neuronal autophagy. In a recent study, we identified that the N-terminal LIR of RavZ is crucial for substrate recognition and cleavage [8]. Additionally, we confirmed that arranging two consecutive LIRs enhances selectivity for LC3/GABARAP-containing autophagosomes in sensor development [9].

**Fig. 1:**
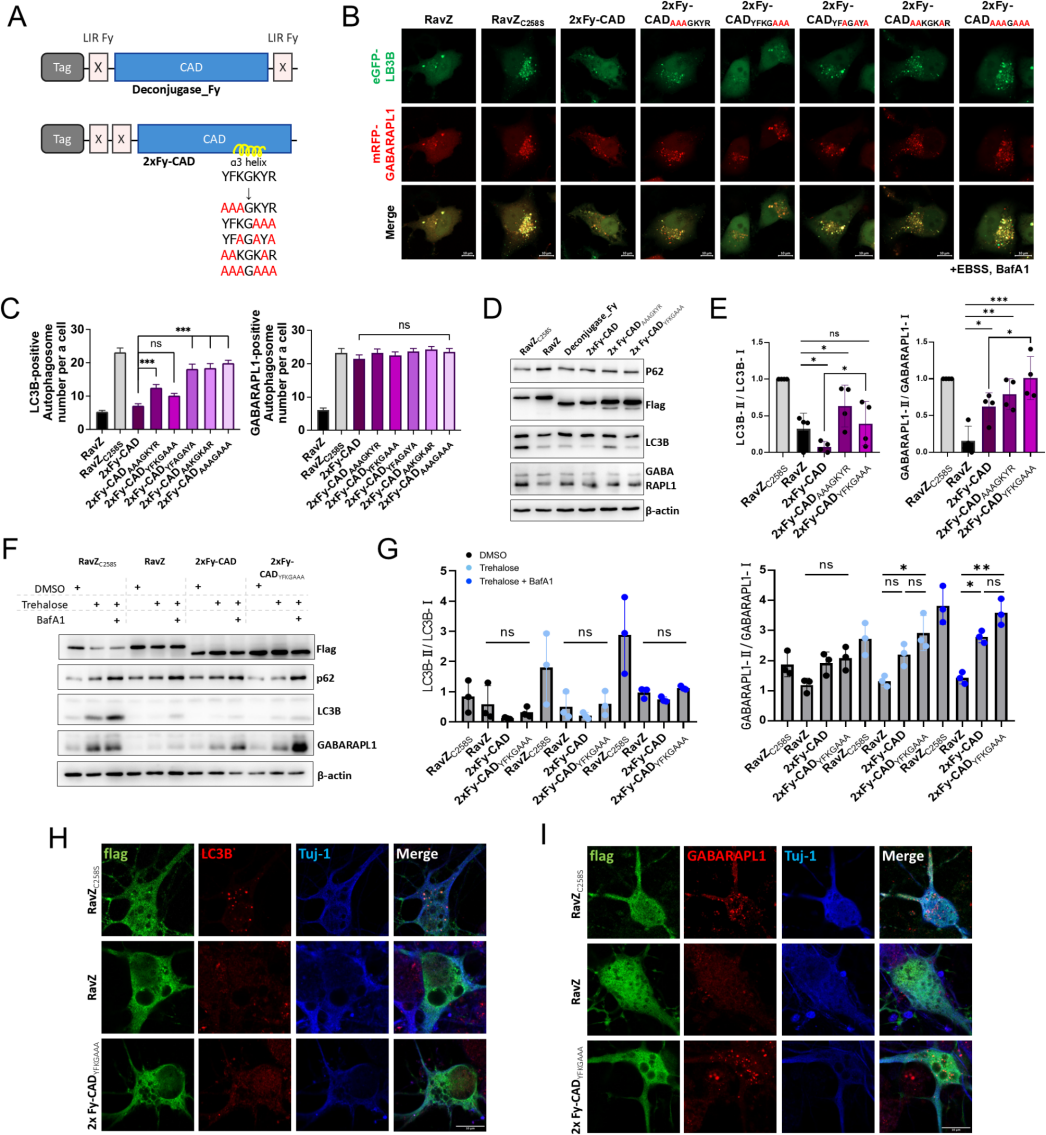
The effect of mutagenesis that partially disrupts the α3-helix structure of the CAD of RavZ on selective enzymatic activity. **(A)** Schematic diagram of 3xFlag-fused deconjugase_Fy and 2xFy-CAD(α3 helix Alanine mutants) constructs. **(B)** Confocal images showing autophagosome formation of eGFP-LC3B and mRFP-GABARAPL1 co-expressed with RavZ proteins or LIR(Fy)-containing α3 helix Alanine mutants of catalytic domain in HKO HeLa cells upon autophagy induction (100 nM bafilomycin A1 [BafA1]) in Earle’s Balanced Salts solution (EBSS) for 2 h, (+ EBSS, BafA1). Scale bar: 10 μm. **(C)** Bar graphs illustrate the each eGFP-LC3B- or mRFP-GABARAPL1-positive autophagosome spot number for each cell. Values are presented as means + SEM (*n* ≥ 20 for each group). ns. not significant; ***P < 0.001 compared with 2xFy-CAD-expressing cells with one-way ANOVA in conjunction with the Newman–Keuls multiple comparison test. **(D)** Western blot showing the delipidation of endogenous LC3B and GABARAPL1 in mouse cortical neuron lysates expressing RavZ proteins or LIR(Fy)-containing α3 helix Alanine mutants of catalytic domain upon autophagy inhibition (50µM CQ for 2 h). **(E)** Bar graphs illustrate the ratios of LC3B-II/LC3B-I and GABARAPL1-II/GABARAPL1-I. Values are presented as means ± SEM (*n* = 4). ns. not significant; *P < 0.033, **P < 0.002, **P < 0.001 compared with RavZ-expressing cells using mixed-effects analysis in conjunction with the uncorrected Fisher’s LSD test. **(F)** Western blot showing the delipidation of endogenous LC3B and GABARAPL1 in mouse cortical neuron lysates expressing RavZ proteins or LIR(Fy)-containing α3 helix Alanine mutants of catalytic domain upon autophagy induction and inhibition (Trahalose 100mM/24 h, bafilomycin A1 10 nM/24 h). **(G)** Bar graphs illustrate the ratios of LC3B-II/LC3B-I and GABARAPL1-II/GABARAPL1-I. Values are presented as means ± SEM (*n* = 3). ns. not significant; *P < 0.033, **P < 0.002 compared with RavZ expressing cells with Friedman test in conjunction with the Dunn’s multiple comparisons test. **(H-I)** confocal images showing formation of LC3B (H) and GABARAPL1 **(I)**-containing autophagosomes in Tuj-1 positive mouse cortical neurons transiently expressing RavZ proteins or 2xFy-CAD(YFKGAAA) upon autophagy inhibition (100nM BafA1 for 2 h). Scale bar: 10 μm

To investigate the functional contribution of the N-terminal LIRs and the α3 helix of the catalytic domain (CAD) in RavZ, we generated 2xFy-CAD by arranging the N-terminal LIRs sequentially and deleting the C-terminal LIR (Fig. 1A). The α3 helix, previously reported to mediate membrane association via weak hydrophobic interactions, was analyzed to determine its role in LC3/GABARAP-II delipidation and its synergy with the N-terminal LIRs [10, 11]. To reduce hydrophobicity and evaluate its impact, several α3 helix mutants were created by substituting specific amino acids with alanine, including YFKGKYR to AAAGAAA, YFAGAYA, AAKGKAR, AAAGKYR, or YFKGAAA.

These 2xFy-CAD variants were expressed in HKO HeLa cells, and their effects on the formation of autophagosomes containing overexpressed LC3B and GABARAPL1 were evaluated. Interestingly, both 2xFy-CAD(AAAGKYR) and 2xFy-CAD(YFKGAAA), similar to RavZ(C258S), a catalytically inactive mutant of RavZ, did not affect the formation of GABARAPL1-containing autophagosomes. However, 2xFy-CAD(AAAGKYR) failed to inhibit LC3B-containing autophagosome formation, indicating insufficient functionality (Fig. 1B, C).

Next, we tested whether 2xFy-CAD(YFKGAAA) could inhibit LC3A/B-containing autophagosome formation in neuronal cells. Mouse cortical neurons were transfected with 2xFy-CAD(YFKGAAA), and western blot and confocal microscopy were used to evaluate LC3B and GABARAPL1 cleavage and autophagosome formation. Neurons were treated with 50 µM chloroquine (CQ) to block autophagosome-lysosome fusion, increasing LC3/GABARAP-II levels and allowing enzyme specificity to be assessed (Fig. 1D, E). The results showed that 2xFy-CAD(YFKGAAA) selectively delipidated endogenous LC3B compared to deconjugase-Fy and 2xFy-CAD. Moreover, upon autophagy induction and inhibition (Trahalose 100mM/24 h, bafilomycin A1 10 nM/24 h), 2xFy-CAD(YFKGAAA) specifically inhibited LC3B-containing autophagosome formation in mouse cortical neurons (Fig. 1F, G). Statistical analysis revealed that in Figs. 1E and 2xFy-CAD(YFKGAAA) exhibited a significantly reduced selectivity for GABARAPL1 compared to 2xFy-CAD (*P* < 0.033, mixed-effects analysis). In Fig. 1G, while statistical significance was not achieved due to the limited sample size (*n* = 4), a consistent trend showed a weaker effect of 2xFy-CAD(YFKGAAA) on GABARAPL1-II conversion. These results suggest that 2xFy-CAD(YFKGAAA) selectively modulates interactions with GABARAPL1 as intended, highlighting the need for further validation. Indeed, in mouse cortical neurons, 2xFy-CAD(YFKGAAA) could selectively inhibit the formation of LC3B-containing autophagosomes while not affecting the formation of GABARAPL1-containing autophagosomes (Fig. 1H, I). These findings confirm the enhanced selectivity and functionality of 2xFy-CAD(YFKGAAA) for LC3A/B in neuronal autophagy.

Our findings highlight the importance of the N-terminal LIR motifs and the α3 helix in modulating the specificity and functionality of RavZ-derived deconjugases in autophagy. By sequentially arranging N-terminal LIRs and mutating the α3 helix to reduce hydrophobicity, we demonstrated that the 2xFy-CAD(YFKGAAA) variant selectively delipidates LC3B without affecting GABARAP-containing autophagosomes. This specificity is particularly significant in neuronal autophagy, where LC3A/B-mediated processes are critical for maintaining cellular homeostasis and other neuronal functions. This study highlights that 2xFy-CAD(YFKGAAA) exhibits high specificity for LC3A/B compared to GABARAPL1, emphasizing its utility in analyzing LC3A/B-mediated pathways. However, its interactions with structurally similar proteins such as LC3C and GABARAPL2 have not been tested in this context. These proteins are known to participate in distinct autophagic and membrane-related processes, and further investigation into their potential interactions with 2xFy-CAD(YFKGAAA) is required. Such studies will refine the tool’s specificity and expand its applicability in diverse autophagy-related research. The successful inhibition of LC3A/B-containing autophagosome formation by 2xFy-CAD(YFKGAAA) suggests its utility as a precise tool to dissect LC3A/B functions in various cellular contexts, particularly in post-mitotic neurons. This tool offers a breakthrough in studying the distinct roles of membrane-anchored LC3 proteins, overcoming the limitations of previous approaches that lacked selectivity for lipidated LC3/GABARAP forms. These results open new avenues for studying autophagy regulation and its implications in neurodegenerative diseases. The ability of 2xFy-CAD(YFKGAAA) to selectively target LC3A/B provides a framework for developing therapeutic strategies aimed at modulating autophagy in a subtype-specific manner. This study’s LC3A/B-specific approach establishes a valuable foundation for investigating autophagy’s role in neurodegenerative diseases, such as Alzheimer’s and Parkinson’s. Future integration of human-derived models, including patient-specific iPSCs and brain organoids, could provide a more physiologically relevant context to study disease-specific autophagy mechanisms. These models also enable the exploration of LC3A/B-mediated processes in disease progression and offer a robust platform for high-throughput therapeutic screening. Such advancements would bridge fundamental research with translational applications, significantly enhancing the clinical relevance of these findings. Additionally, engineering similar tools with enhanced specificity for other LC3/GABARAP proteins could facilitate a deeper understanding of autophagy’s diverse functions across different cell types and conditions. This work underscores the potential of tailored deconjugases as both research tools and therapeutic agents in autophagy-related disorders.

## Data Availability

No datasets were generated or analysed during the current study.

## Abbreviations

LC3: microtubule associated protein 1 A/1B-light chain 3
GABARAP: Gamma-aminobutyric acid receptor-associated protein
ATG: Autophagy-related protein
PE: Phosphatidylethanolamine
LIR: LC3-interacting region
CAD: Catalytic domain of RavZ
Fyco1 or Fy: FYVE and coiled-coil domain–containing protein 1
BafA1: Bafilomycin A< Subscript>1</Subscript>
EBSS: Earle’s Balanced Salts solution
CQ: Chloroquine
